# Ecological interactions shape the adaptive value of plant defence: Herbivore attack versus competition for light

**DOI:** 10.1111/1365-2435.13234

**Published:** 2018-11-20

**Authors:** Jorad de Vries, Jochem B. Evers, Marcel Dicke, Erik H. Poelman

**Affiliations:** ^1^ Laboratory of Entomology Wageningen University Wageningen The Netherlands; ^2^ Centre for Crop System Analysis Wageningen University Wageningen The Netherlands

**Keywords:** *Brassica nigra*, competition, ecological costs, functional‐structural plant modelling, growth‐defence trade‐off, herbivory, herbivore interactions, plant, plant defence

## Abstract

1. Plants defend themselves against diverse communities of herbivorous insects. This requires an investment of limited resources, for which plants also compete with neighbours. The consequences of an investment in defence are determined by the metabolic costs of defence as well as indirect or ecological costs through interactions with other organisms. These ecological costs have a potentially strong impact on the evolution of defensive traits, but have proven to be difficult to quantify.
2. We aimed to quantify the relative impact of the direct and indirect or ecological costs and benefits of an investment in plant defence in relation to herbivory and intergenotypic competition for light. Additionally, we evaluated how the benefits of plant defence balance its costs in the context of herbivory and intergenotypic competition.
3. To this end, we utilised a functional‐structural plant (FSP) model of *Brassica nigra* that simulates plant growth and development, morphogenesis, herbivory and plant defence. In the model, a simulated investment in defences affected plant growth by competing with other plant organs for resources and affected the level and distribution of herbivore damage.
4. Our results show that the ecological costs of intergenotypic competition for light are highly detrimental to the fitness of defended plants, as it amplifies the size difference between defended and undefended plants. This leads to herbivore damage counteracting the effects of intergenotypic competition under the assumption that herbivore damage scales with plant size. Additionally, we show that plant defence relies on reducing herbivore damage rather than the dispersion of herbivore damage, which is only beneficial under high levels of herbivore damage.
5. We conclude that the adaptive value of plant defence is highly dependent on ecological interactions and is predominantly determined by the outcome of competition for light.

plain language summary is available for this article.

## 1 INTRODUCTION

In natural settings, plants are part of complex communities of herbivores and neighbouring plants that shape the adaptive value of growth and defence‐related traits (de Vries, Evers, & Poelman, 2017; Lankau & Strauss, 2008; Poelman, 2015). The interactions within these communities give rise to trade‐offs in growth and defence that maximise fitness while responding to a variable environment (Züst & Agrawal, 2017). Direct competition between two plant traits over the limiting pool of an individual's internal resources is perhaps the most commonly considered driver of the trade‐offs observed between a plant's ability to defend against herbivorous insects and the mechanisms that allow the plant to compete for light with neighbouring plants (Ballare, 2014; Herms & Mattson, 1992; Züst & Agrawal, 2017; Züst, Joseph, Shimizu, Kliebenstein, & Turnbull, 2011). It is apparent that defensive mechanisms bring substantial metabolic costs that include costs of the machinery for the synthesis, modification, transport, maintenance and storage of the plant secondary metabolites (Bekaert, Edger, Hudson, Pires, & Conant, 2012; Gershenzon, 1994). However, these direct costs do not always result in a loss of fitness and might only be relevant under certain ecological settings such as resource limitation, competition for resources, the presence of herbivores and pathogens or when the plant's mutualists are affected (Cipollini, Walters, & Voelckel, 2014; Dicke & Hilker, 2003; Heil & Baldwin, 2002; Koricheva, 2002; Strauss, Rudgers, Lau, & Irwin, 2002). The expression of costs through interactions between the plant and biotic or abiotic conditions in its environment can be defined as ecological costs. These ecological costs can have a substantial impact on plant fitness and are, therefore, important drivers of evolution (Dicke & Hilker, 2003; Heil, 2002). However, identifying and quantifying the ecological costs associated with plant defence is complicated by the myriad of possible effects of the plant defence trait on other community members (Heil & Baldwin, 2002; Stam et al., 2014). Even when isolating a single interaction in an experimental set‐up, discriminating ecological costs from metabolic costs is often complicated due to the complex and interwoven nature of the physiological and ecological mechanisms driving plant–plant–herbivore interactions (de Vries et al., 2017).

The interaction between physiological and ecological costs is apparent in the synthesis and allocation of plant chemical defences against insect herbivores. Plants are known to exhibit a stronger defence response in younger leaves (Koricheva & Barton, 2012), which follows the allocation of key nutrients such as nitrogen towards plant parts that are most favourably positioned relative to resource gradients (Hikosaka et al., 2016; Hirose, 2005; Hirose, Werger, Pons, & Rheenen, 1987; McKey, 1974). This local pattern of defence expression offers potential benefits to the plant if it results in dispersing herbivore damage within the plant and away from most valuable tissues (Cipollini et al., 2014). However, the response of a herbivore to a plant's defence expression depends on that herbivore's sensitivity to plant taxon‐specific secondary metabolites, which differs greatly between herbivore species (Bennett & Wallsgrove, 1994). Those that have specialised on a particular host‐plant taxon are more resistant to the defensive mechanisms adopted by that taxon, making them less susceptible to the toxic or digestive reducing function of the defensive secondary metabolite. This differentiation in host‐plant specialisation makes the composition of the insect community attacking the plant a strong determinant of the benefits the plant receives for its investment in defence. Heterogeneity in the distribution of nutritional and defensive value of leaves in the canopy is expected to result in different herbivore distribution patterns depending on the level of specialisation of the members of the herbivore community. The increased resistance of specialised herbivores to defensive compounds allows them to feed from the more nutritious, yet better defended parts of their host plant such as young leaves, buds and seeds (Cates, 1980; Feeny, 1976; Schoonhoven, van Loon, & Dicke, 2005). Conversely, the elevated levels of defence in these important plant parts deter the more generalist herbivore species that are then forced to feed on less defended but also less nutritional plant tissues such as mature leaves (Cates, 1980; Feeny, 1976; Schoonhoven et al., 2005). The distribution pattern of the herbivore community resulting from these differences in herbivore sensitivity to plant defence in turn has a strong impact on plant fitness. This is especially true in a competitive environment where the removal of young leaves decreases plant competitive ability and consequentially fitness more than the removal of mature leaves (de Vries, Poelman, Anten, & Evers, 2018). Therefore, we expect the adaptive value of plant defence to be more dependent on the ecological costs through the effect on the plant's competitive ability and herbivore damage than on the metabolic costs of these defences (Agrawal, 2000; Heil, Hilpert, Kaiser, & Linsenmair, 2000; Van Dam & Baldwin, 2001).

In this paper, we aim to (a) quantify the direct costs of plant defence as well as the ecological costs imposed by herbivore damage and intergenotypic competition for light. We expect the ecological costs imposed by herbivore damage and intergenotypic competition for light to exceed the direct costs of plant defence, resulting in a stronger impact on plant fitness. We also aim to (b) quantify the direct benefits of plant defence through a reduction or redistribution of herbivore damage, as well as the ecological effect of this benefit under intergenotypic competition for light. Finally (c), we evaluate the level of benefits required to outweigh the direct and ecological costs of plant defence, at which point plant defence becomes adaptive to the plant. We expect plant defence to be especially effective if it results in dispersion as well as reduction of herbivore damage. Here, we study the interaction between defence investment, competition for light and herbivory using a modelling approach called functional‐structural plant (FSP) modelling (Evers, 2016; Vos et al., 2010). This three‐dimensionally explicit modelling approach allows for the simulation of individual plants that grow and compete for resources with neighbouring plants. Functional‐structural plant modelling has proven to be a powerful tool to simulate plant competition for light and the associated effects on source‐sink dynamics (Evers & Bastiaans, 2016; Evers et al., 2010) and architectural responses (Bongers, Pierik, Anten, & Evers, 2018; Evers et al., 2007; Zhu, Werf, Anten, Vos, & Evers, 2015).

## 2 MATERIALS AND METHODS

### 2.1 Model description

To elucidate the interaction between plant competition for light, herbivory and plant defence, we expanded the plant–herbivore FSP model of *Brassica nigra* described previously (de Vries et al., 2018), which is developed in the modelling platform GroIMP (Hemmerling, Kniemeyer, Lanwert, Kurth, & Buck‐Sorlin, 2008). This model has been parameterised and validated using detailed field measurements on *B. nigra* architecture, growth and development, biomass and seed yield. In summary, this three‐dimensional model mechanistically simulates aboveground plant growth and competition for light through source‐sink dynamics in carbon assimilation and allocation and light‐driven mediation of plant architecture (for a detailed model description, see de Vries et al. (2018)). We expanded the existing model by adding a plant defence module that impacts herbivore damage and acts as a carbon sink, interacting with plant growth through the source‐sink dynamics of the plant. These additions are described in detail below.

In the model, we simplified plant defence to an on/off mechanism where defended plants allocate a fixed percentage (*D*) of the assimilates produced with photosynthesis (*A*_total_, in g) towards the biosynthesis and maintenance of plant defence. The remaining assimilates (*A*_growth_, in g) are allocated to the maintenance of standing biomass and the growth of new biomass.

(1) $$A_{\text{growth}}=\left(1-\frac{D}{100}\right)*A_{\text{total}}$$

**Figure 1:**
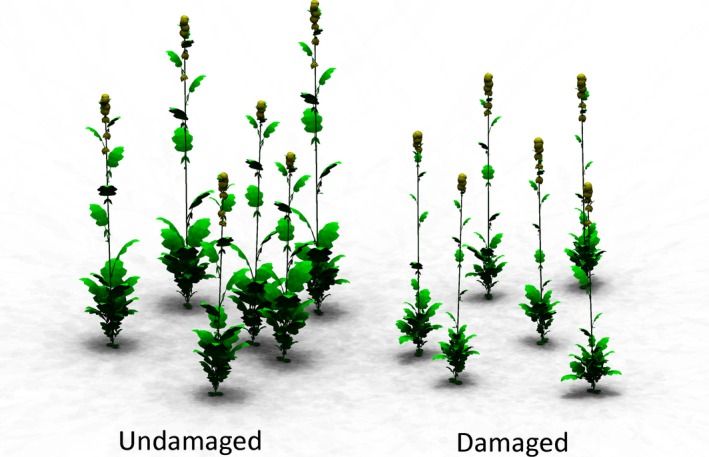
Visual representation of the functional‐structural plant (FSP) model, showing undamaged and damaged plants. Herbivore damage reduces leaf area, which affects canopy structure and subsequent light climate. For visualisation purposes, the plant density in the figure is lower than the plant density used in the simulations

In the model, herbivory is represented by a rate of damage to an individual leaf over time as a function of leaf area (Feeny, 1976; Johnson & Agrawal, 2005; Schoonhoven et al., 2005), capturing the damage done by a community of chewing herbivores. This rate of herbivore damage reduces both the current size of a leaf, representing the actual removal of area, and the potential size of a leaf, limiting the further growth of a damaged leaf (see Figure 1 for the results of herbivore damage on plant architecture). The rate of herbivore damage is fixed on the plant level, assuming no dispersal of herbivores between plants as a result of a plant's defence investment. The herbivore damage suffered by a plant is simulated using a sigmoidal function that describes the total amount and distribution of herbivore damage within the plant (Equation (2)). The rate of damage by herbivory on the leaf level per growing degree day (GDD) (dmg, g leaf biomass/GDD) is calculated using the leaf biomass (b, in g), the relative leaf rank (*r*), the herbivore distribution (*h*, value from 0 to 1, see Figures 3 and 4.), the base rate of leaf removal (*c*, fraction of leaf biomass GDD^−1^) and the damage reduction by plant defence (*d*, %):

(2) $$\text{dmg =}\;\frac{c*\left(1-\frac{d}{100}\right)*b}{1+\exp\left(10*\left(h-\left(1-h\right)\right)*\left(r-0.5\right)\right)}$$

Leaf rank was used to number the leaves and is an indicator of the leaf's position along the main stem. The relative rank of a leaf (*r*) is calculated using the absolute rank of the leaf (*r*_a_), the highest (*r*_max_) and lowest (*r*_min_) ranked leaf on the same plant:

(3) $$r=\frac{r_{a}-r_{\text{min}}}{r_{\text{max}}-r_{\text{min}}}$$

Equation (2) was simplified for undefended plants where the damage reduction by plant defence equals 0 and the herbivore distribution parameter equals 0.2, which represents herbivore preference for young leaves in the absence of defence (Cates, 1980; Schoonhoven et al., 2005):

(4) $$\text{dmg}=\frac{c*b}{1+\exp\left(-6*\left(r-0.5\right)\right)}$$

This function allows for simulation of different scenarios of costs and benefits of plant defence, which are described in the next section. Plant defence affects the total amount of herbivore damage, describing the reduction of herbivore growth and the subsequent reduction of herbivore damage. The model does not explicitly describe defence expression at the leaf level, but implicitly assumes that the presence of defence can affect the distribution of herbivore damage (depending on the scenario), reflecting how different herbivore species in the herbivore community might respond to plant defence. In undefended plants, we assume a distribution of herbivore damage that favours young leaves due to these being more nutritional than older leaves (which is not explicitly represented in the model). In defended plants, we expect a dispersal of generalist herbivore species towards older leaves (Cates, 1980; Schoonhoven et al., 2005), which is modelled through a shift in the shape of the sigmoidal function described in Equation (2) by increasing the value of *h*.

### 2.2 General simulation set‐up

Plants were simulated in plots of 16 plants at a density of 100 plants/m^−2^. This small plot was cloned 625 times to construct a large field with 10,000 plants for light model calculations, where every individual plant is represented 625 times at regular intervals. The light intercepted during a time step by an individual plant is calculated as the average light interception of its clones. This method evens out border effects that would otherwise be prevalent in a small plot as the the clones of an individual plant occupy a large variety of locations both close to and far away from the borders of the field. Simulations ran from the 31st of March to the 2nd of August (124 days), with average daily temperature, average daily insolation and solar angle typical for the Netherlands at a latitude of 52 degrees (Evers et al., 2010; Zhu et al., 2015).

### 2.3 Scenarios: Direct and ecological costs of plant defence (i)

To quantify the impact of plant defence on plant fitness, we imposed five levels of photosynthetic costs on defending plants (*D* = 5%, 10%, 15%, 20% and 25% of assimilates produced by photosynthesis in Equation (1)), which spans the range of direct costs found in a multitude of plant species (Bekaert et al., 2012; Strauss et al., 2002).

The treatments were set up as follows:

1. We first simulated monostands of undefended, undamaged plants that act as a control, providing a baseline measure of plant fitness to which the following treatments are compared.
2. To quantify the direct (metabolic) costs of plant defences, we simulated monostands of defended plants in the absence of herbivore damage.
3. To quantify the ecological costs of herbivore damage, we simulated monostands of plants that invested in defence and suffered low (*c* = 0.005) or high (*c* = 0.01) herbivore damage, without receiving benefits for their investment in defence (*h* = 0.2, *d* = 0, Equation (2)).
4. To quantify the ecological costs imposed by intergenotypic competition, we simulated mixed stands of defended and undefended plants in the absence of herbivore damage.
5. To quantify the combined effect of herbivore damage and intergenotypic competition, we then simulated mixed stands of defended and undefended plants in which all plants suffered low (*c* = 0.005) or high (*c* = 0.01) herbivore damage, without the defending plants receiving a benefit for their investment in defence (*h* = 0.2, *d* = 0, Equation (2)).

### 2.4 Scenarios: Direct and ecological benefits of plant defence (ii)

To quantify the direct and ecological benefits of plant defence, we simulated defended plants that did not pay the metabolic costs associated with this defence investment. These plants were simulated in monostands to determine the direct benefits of plant defence and in mixtures to determine the ecological benefits of plant defence. Both the monostands and mixtures were subjected to low (*c* = 0.005) or high (*c* = 0.01) herbivore damage, and we simulated six levels of herbivore damage reduction for defended plants (*d* = 0, 10, 20, 30, 40, 50%, Equation (2)) and three herbivore distributions for defended plants (*h* = 0.2, 0.5, 0.8, Equation (2)) in a full factorial design.

### 2.5 Scenarios: Costs and benefits of plant defence (iii)

To quantify when the benefits of plant defence outweigh the total costs of plant defence, we simulated mixtures of defended and undefended plants where all plants suffered low (*c* = 0.005) or high (*c* = 0.01) herbivore damage and where the defended plants allocated 15% of the assimilates produced by photosynthesis to defence (Bekaert et al., 2012). We assumed that plant defence can reduce damage as well as change the distribution of damage within the plant from younger towards older leaves. To quantify the importance of reducing the total amount of damage and the distribution of damage, we simulated six levels of herbivore damage reduction for defended plants (*d* = 0, 10, 20, 30, 40, 50%, Equation (2)) and tree herbivore distributions for defended plants (*h* = 0.2, 0.5, 0.8, Equation (2)) in a full factorial design. The undefended plants suffered the baseline level of herbivore damage (*d* = 0), the distribution of which was skewed towards young leaves (*h* = 0.2).

### 2.6 Output

The simulated seed yield (e.g. the investment of biomass into seeds) per plant was used as a proxy for plant fitness. Seed yield is an emergent property of the model that arises from the interaction between source‐sink dynamics, herbivore damage and competition for light. We use one of three types of output to show our results:

1. To quantify the direct costs of plant defences, we simulated monostands of undefended, undamaged control plants to act as a baseline for plant fitness. To quantify the ecological costs of plant defences, we simulated mixtures of defended and undefended plants, using the undefended plants to act as a baseline for the fitness of the defended plants. We calculate the costs imposed by a given treatment (C, % yield decrease) by comparing the yield of the treatment (Yield_T_) to the baseline yield of the control plants (Yield_C_).
  (5) $$C=\left(1-\frac{{\text{Yield}}_{T}}{{\text{Yield}}_{C}}\right)*100$$

- To quantify the direct benefits of plant defences, we simulated monostands of undefended plants facing low or high herbivore damage to act as a baseline for plant fitness. To quantify the ecological benefits of plant defences, we simulated mixtures of defended and undefended plants facing low or high herbivore damage and used the undefended plants to act as a baseline for the fitness of the defended plants. We calculate the benefits provided by a given treatment (B, % yield increase) by comparing the yield of the treatment (Yield_T_) to the baseline yield of the control plants (Yield_C_).

(6) $$B=\left(\frac{{\text{Yield}}_{T}}{{\text{Yield}}_{C}}-1\right)*100$$

The model output was tested for significance at the 5% probability level by conducting an analysis of variance (ANOVA). Values reported in the text are shown as (mean ± *SE* unit), and error bars in graphs represent the standard error of the mean.

## 3 RESULTS

### 3.1 Direct and ecological costs of plant defence (i)

**Figure 2:**
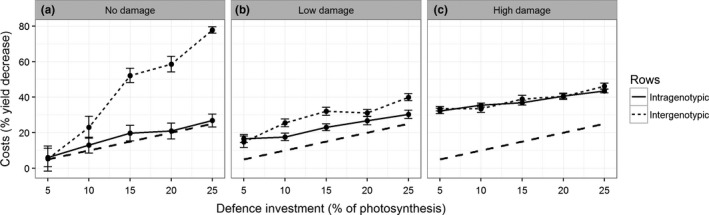
The costs (% yield decrease, *y*‐axis) imposed by five levels of defence investment (% of photosynthesis, *D* in Equation (1), *x*‐axis) for defended plants facing intragenotypic competition with other plants of the same defence type (solid line) or intergenotypic competition with undefended plants (dotted line) and either no (a), low (b) or high (c) herbivore damage. The dashed line represents the line where the yield decrease is proportional to the investment in plant defence. Error bars show standard error of the mean

The direct effect of investing in plant defence on plant fitness was proportional to the percentage of photosynthesis that was invested in defence (Figure 2a). Intergenotypic competition with undefended plants had a disproportionately strong negative effect on the yield of defended plants in the absence of herbivory (*F* = 64.9, *p* < 0.001; Figure 2a). The direct effect of herbivore damage decreased with an increasing investment in defence (difference between the solid/dotted line and the dashed line in Figure 2b,c, *F* = 5.0, *p* < 0.05). This decrease in the effect of herbivory was caused by a reduction of plant size with an increasing investment into defence in conjunction with herbivore damage being proportional to leaf area. This mechanism also led to herbivore damage reducing the negative effect of intergenotypic competition (*F* = 27.6, *p* < 0.001; Figure 2b,c). Herbivore damage balanced the yield differences between defended and undefended plants that emerged from intergenotypic competition, as competitively strong plants suffered more herbivore damage due to their larger size. Intergenotypic competition still reduced the yield of defended plants when suffering low rates of herbivore damage (*F* = 13.7, *p* < 0.001; Figure 2b), but had no effect on the yield of defended plants under high rates of herbivore damage (*F* = 0.9, *p* = 0.35; Figure 2c).

### 3.2 Benefits of plant defence (ii)

**Figure 3:**
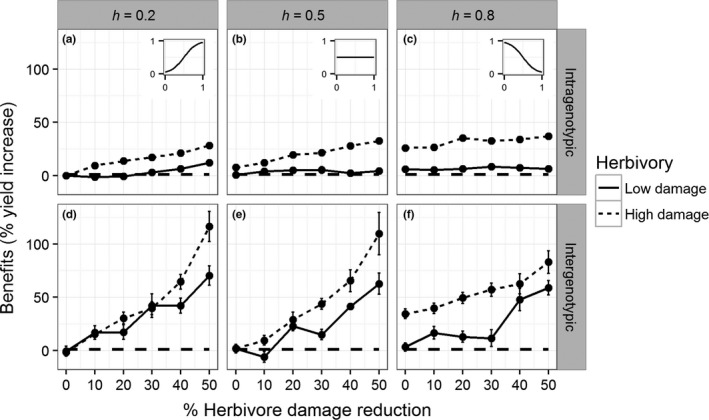
The benefits (% yield increase, *y*‐axis) as a result of a reduction in herbivore damage (*x*‐axis) or a redistribution of herbivore damage (columns, h in Equation (3) for defended plants that payed no costs for their defence investment and faced either intragenotypic competition with other defended plants (a,b,c) or intergenotypic competition with undefended plants (d,e,f). The dashed horizontal line represents the level above which the defended plants out‐compete undefended plants. The subplots show the herbivore distribution (*y*‐axis) as a function of relative leaf rank (*x*‐axis, lowest leaf rank = 0, highest leaf rank = 1) for the corresponding value of h. Error bars show standard error of the mean

The direct benefits of plant defence, illustrated by a plant facing intragenotypic competition, were apparent both when reducing (*F* = 53.7, *p* < 0.001; Figure 3a,b,c) and when redistributing (*F* = 25.6, *p* < 0.001; Figure 3a,b,c) herbivore damage, but the fitness benefits were far more substantial under high than under low levels of herbivore damage (*F* = 323, *p* < 0.001; Figure 3a,b,c). In plants that faced low levels of herbivore damage and intergenotypic competition with undefended plants, these direct benefits translated to an indirect benefit when reducing herbivore damage (*F* = 148, *p* < 0.001; Figure 3d,e,f), but not when redistributing herbivore damage (*F* = 2.2, *p* = 0.11; Figure 3d,e,f). Under high levels of herbivore damage, the direct benefits translated to an indirect benefit both when reducing herbivore damage (*F* = 210, *p* < 0.001; Figure 3d,e,f) and when redistributing herbivore damage (*F* = 3.1, *p* < 0.05; Figure 3d,e,f).

### 3.3 Costs versus benefits of plant defence (iii)

**Figure 4:**
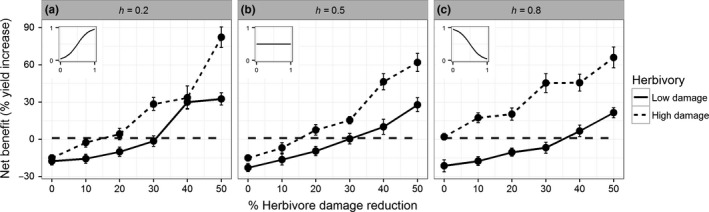
The net benefit (*y*‐axis, % yield increase) provided by a defence investment while competing with undefended plants competing in mixed stands. The defended plants benefitted from their defences by reducing herbivore damage (*x*‐axis, % herbivore damage reduction, d in Equation (2) and/or changing the distribution of herbivore damage (panels, h in Equation (3). The dashed horizontal line represents the level above which the defended plants out‐compete their undefended neighbours. The subplots show the herbivore distribution (*y*‐axis) as a function of relative leaf rank (*x*‐axis, lowest leaf rank = 0, highest leaf rank = 1) for the corresponding value of h. Error bars show standard error of the mean

To quantify the level of benefits required to balance the investment costs of plant defence, we simulated mixed stands of defended and undefended plants in which plant defence changed the distribution and/or amount of herbivore damage, assuming a damage investment percentage of 15%. Our results show that under low levels of herbivory, defended plants out‐competed their undefended neighbours, resulting in a positive net benefit, when their defence resulted in at least a 30% reduction in herbivore damage (Figure 4). Under high levels of herbivory, defended plants out‐competed their undefended neighbours, resulting in a positive net benefit, when their defence resulted in at least a 10% reduction in herbivore damage (Figure 4). Alternatively, when the presence of defence drove herbivores away from young leaves, skweing the herbivore distribution towards old leaves, the defending plants out‐competed their undefended neighbours regardless of the herbivore damage reduction (h=0.8, Figure 4c). These tipping points cannot be explained by the direct costs and benefits of defence as the fitness decrease as a result of a defence investment (15%) was higher than the direct fitness benefit of reducing herbivore damage at the tipping points (low herbivory: 2.7%–8.5% yield increase at 30% damage reduction; high herbivory: 9.2%–12% yield increase at 10% damage reduction, see Figure 3). The differences in herbivore distributions did not lead to differences in plant fitness under low levels of herbivore damage (*p* = 0.43, Figure 4), but could lead to an increase in plant fitness under high levels of herbivore damage (*F* = 9.5, *p* < 0.005, Figure 4). These differences in herbivore distribution did affect the plant, as shown by the final biomass of leaves, which was affected by the herbivore distribution such that the final leaf biomass was inversely correlated with herbivore damage distribution (Supporting Information Figure S1).

## 4 DISCUSSION

We show that the indirect costs and benefits of plant defence through ecological interactions are more important than, and disproportionate to, the effects of direct costs and benefits of plant defence on plant fitness. Our results show that the direct costs of a defence investment were proportional to the size of the investment. However, the indirect effects through ecological interactions with herbivores and neighbouring competitors were highly context dependent and not proportional to the direct effect on plant fitness. Our results further show that the ecological costs of intergenotypic competition had the strongest impact on plant fitness among the tested treatment (Figure 2). The ecological costs of competition for light scaled disproportionately with the investment in plant defences, which was caused by the asymmetric nature of competition for light (e.g. stronger competitors taking a disproportionate share of resources (Weiner, 1990; Freckleton & Watkinson, 2001)). The model assumed that herbivore damage scaled with leaf area, making it dependent on plant size (Feeny, 1976; Johnson & Agrawal, 2005; Schoonhoven et al., 2005). This led to a decrease in the effect of herbivore damage on yield with an increasing investment in defence (difference between the solid/dotted lines and the dashed lines in Figure 2b,c) and a decrease in the negative effect of a defence investment under intergenotypic competition due to herbivore damage (difference between the dotted lines in Figure 2a–c). If we were to assume that herbivore damage did not scale with leaf area and remained constant regardless of plant size, herbivore damage would likely amplify the asymmetry of competition for light. In this case, the amount of damage inflicted by the herbivores relative to plant size is higher for under‐performing plants, giving them a further disadvantage compared to their over‐performing neighbours.

Our results show that the dispersion of herbivore damage from young leaves to older leaves netted the defended plants a fitness benefit only under high levels of herbivore damage (Figures 3 and 4). An earlier study addressing an isolated plant–herbivore interaction rather than the aggregated effect of an entire herbivore community found that damage to young leaves was more detrimental to plant fitness than damage to old leaves (de Vries et al., 2018). This earlier study simulated severe herbivore damage, as the herbivore damage was concentrated in a small period of time rather than spread over the entire development of the plant. This shows that the isolated effect of a single plant–herbivore interaction at a given point during the plant's development can be very different from the aggregated effect of an average herbivore community over the entirety of the season. In the context of temporally dispersed rather than temporally concentrated herbivore damage, our results show that the adaptive value of plant defence in a competitive environment relies on reducing herbivore damage rather than dispersing herbivore damage (Figure 4). Generalist herbivores are highly susceptible to plant defence and are, therefore, severely hampered in their growth and survival by taxon‐specific secondary metabolites (Gols et al., 2008; Poelman, Broekgaarden, Loon, & Dicke, 2008). However, most specialist herbivores are mildly hampered by the plant's defence (Poelman et al., 2008; Wei, Vrieling, Mulder, & Klinkhamer, 2015), despite feeding from well defended yet highly nutritional young leaves (Cates, 1980; Feeny, 1976). This leads us to predict that defence is disfavoured when plants are under attack by specialist herbivores as the benefits of defending against a specialist herbivore are less likely to outweigh the costs of the defence investment. This prediction supports data by Lankau (2007), who showed that generalist and specialist herbivores exert opposing selection pressures on plant defence, where high levels of defence are favoured in the absence of specialist herbivores and disfavoured in the absence of generalist herbivores. The negative selection pressure of specialist herbivores is further strengthened by the role of secondary metabolites in food‐plant selection by specialists, making plants with a high level of defence more vulnerable to attack by specialist herbivores (Badenes‐Perez, Gershenzon, & Heckel, 2014; Badenes‐Pérez, Reichelt, Gershenzon, & Heckel David, 2010; Poelman et al., 2008; Poelman, Loon, Dam, Vet, & Dicke, 2010). The study of invasive plant species might shed light on the selective pressure exerted by generalist and specialist herbivores on plant defence as invasive plant species experience herbivore communities that often lack their native specialist herbivores. These invasive plant species show increased competitive ability and are more resistant to generalist herbivores but are less resistant to specialist herbivores compared to their native conspecifics (Lin, Klinkhamer, & Vrieling, 2015). This might indicate that not only the level but also the complexity of plant defence is under different selection by herbivore species with different levels of specialisation (Lankau & Strauss, 2008). A more complex blend of secondary metabolites is costlier for the plant to produce, while the benefits are dependent on the attacking herbivore. As a result, the complexity in secondary metabolites potentially plays an important role in determining the adaptive value of plant defence.

Plants growing in high densities maximise their ability to compete for light through a suite of morphological changes such as increased internode elongation and leaf hyponasty, termed the shade avoidance syndrome (Ballaré & Pierik, 2017; Fraser, Hayes, & Franklin, 2016). These morphological changes are regulated by the ratio of red to far‐red (R:FR) light in the spectrum reflected within a canopy, which is a robust signal of neighbour presence as plant tissues readily absorb red light while the far‐red light is reflected or transmitted (Ballare, Scopel, & Sanchez, 1990). This low R:FR signal also reduces the plant's defensive capabilities by desensitising the plant to jasmonic acid (JA), one of the essential phytohormones that regulate plant defence (Ballare, 2014; Campos et al., 2016; de Wit et al., 2013; Moreno, Tao, Chory, & Ballare, 2009). The mediation of defences by R: FR is indicative of an interactive effect on selection pressure between herbivory and competition, and multiple functions of this mechanism to regulate the plant phenotype have been identified (de Vries et al., 2017). The most obvious function of this mechanism is a resource‐driven trade‐off between the ability of a plant to defend against attackers and its ability to out‐compete its neighbours (Ballare, 2014; Herms & Mattson, 1992), as is apparent when comparing strategies of different plant species. Alternatively, this mechanism could be a means of optimal defence partitioning within the canopy (Izaguirre, Mazza, Astigueta, Ciarla, & Ballaré, 2013; McKey, 1974), increasing defence investment towards leaves that represent the highest investment in current and future resource acquisition. This gradient in defence partitioning could function to disperse herbivores within the canopy, driving generalist herbivores away from younger and more valuable leaves. A third possibility is a mechanism to reduce plant defence expression as a whole, to decrease plant attractiveness to specialist herbivores (Poelman & Kessler, 2016), which are potentially more harmful to plant competitiveness than generalist herbivores (de Vries et al., 2018). Each of these non‐exclusive hypotheses is a potential explanation for and may have contributed to the downregulation of defences by R:FR. Our results have shown that intergenotypic competition for light is a highly impactful driver of plant fitness that potentially plays a strong role in determining the adaptive value of plant defence. Our results also suggest that the most likely function of the downregulation of defences by R:FR lies in reducing overall costs of defence while optimising the benefits provided, rather than shaping the distribution of herbivores within the plant. However, our model does not regard single plant–herbivore interactions, which are more variable in space and time and therefore might have a much stronger impact on the adaptive value of plant defence than suggested by the results of this study (de Vries et al., 2018; Poelman & Kessler, 2016).

In this study, we focussed on one possible ecological interaction in a single ecological setting to highlight the importance of studying these interactions to understand the way plants function. We focussed on *Brassica nigra* as a model plant, which warrants the chosen set of conditions as it often occurs in dense monostands. However, the growing conditions faced by other plant species are likely very different from those of *B. nigra*. We focussed on a single form of competition, for light, while plants compete for a plethora of other critical resources such as water, nitrogen and phosphorous. Knowledge on the physiology and ecology of competition for light is well established (Ballaré & Pierik, 2017), and great progress is being made on the physiology of root architectural responses to nutrient availability (Bisseling & Scheres, 2014) and their effects on nutrient competition (Rasmussen, Weisbach, Thorup‐Kristensen, & Weiner, 2017). This study focusses on a generic plant–herbivore interaction while we know from studies on plant–herbivore communities (Poelman & Kessler, 2016) and the rhizosphere microbiome (Berendsen, Pieterse, & Bakker, 2012; Mommer, Kirkegaard, & van Ruijven, 2016; Philippot, Raaijmakers, Lemanceau, & Putten, 2013) that the individual interactions in these complex communities can be highly species specific yet play a major role in plant performance (Berendsen et al., 2018).

A next step in elucidating how the plant balances growth and defence is to place more emphasis on the temporal aspects of plant–herbivore interactions. The expression of defences on the plant level changes during the development of the plant and is more variable than can be expected based on the ontogenetic defence trajectory at the leaf level (Barton & Boege, 2017). The costs and benefits of plant defence as well as the impact of herbivore damage are all relative to the plant's developmental stage (Boege & Marquis, 2005). Herbivore infestation early in development is potentially far more devastating to plant fitness as well as the plant's ability to out‐compete neighbours than an infestation in later stages of development. Additionally, a herbivore can move to a neighbouring plant during the most voracious stage in its development to avoid both the induced defences and reduced feeding potential of its host plant (Dam & Baldwin, 1998), a dynamic that is not included in the scope of this paper. When assessing the costs and benefits of defences on plant competitiveness, we should consider that the costs are paid at an earlier moment during plant development than the benefits are reaped. The time between these events and the predictability of this time interval are also potentially important drivers of selection towards induced or constitutive defences. These temporal interactions are another potential source of ecological costs in addition to competition for light and infestation with generalist or specialist herbivores, which highlights how the costs and benefits of plant defence are primarily dependent on ecological interactions.

## AUTHOR CONTRIBUTION

J.d.V. designed and coded the model, designed and conducted the simulations and analysed the data. J.d.V., E.H.P. and J.B.E. designed the simulations, and all authors interpreted the data and wrote the manuscript.

## Data Availability

The simulation models used in this study are available via Zenodo; https://doi.org/10.5281/zenodo.1468422 (de Vries, Evers, Dicke, & Poelman, 2018)

## References

[fec13234-bib-0001] Agrawal, A. A. (2000). Benefits and costs of induced plant defense for Lepidium virginicum (Brassicaceae). Ecology, 81, 1804–1813. 10.2307/177272

[fec13234-bib-0002] Badenes‐Perez, F. R. , Gershenzon, J. , & Heckel, D. G. (2014). Insect attraction versus plant defense: Young leaves high in glucosinolates stimulate oviposition by a specialist herbivore despite poor larval survival due to high saponin content. PLoS ONE, 9, e95766 10.1371/journal.pone.0095766 24752069 PMC3994119

[fec13234-bib-0003] Badenes‐Pérez, F. R. , Reichelt, M. , Gershenzon, J. , & Heckel David, G. (2010). Phylloplane location of glucosinolates in Barbarea spp. (Brassicaceae) and misleading assessment of host suitability by a specialist herbivore. New Phytologist, 189, 549–556. 10.1111/j.1469-8137.2010.03486.x 21029103

[fec13234-bib-0004] Ballare, C. L. (2014). Light regulation of plant defense. Annual Review of Plant Biology, 65, 335–363. 10.1146/annurev-arplant-050213-040145 24471835

[fec13234-bib-0005] Ballaré, C. L. , & Pierik, R. (2017). The shade‐avoidance syndrome: Multiple signals and ecological consequences. Plant, Cell & Environment, 40, 2530–2543. 10.1111/pce.12914 28102548

[fec13234-bib-0006] Ballare, C. L. , Scopel, A. L. , & Sanchez, R. A. (1990). Far‐red radiation reflected from adjacent leaves: An early signal of competition in plant canopies. Science, 247, 329–332. 10.1126/science.247.4940.329 17735851

[fec13234-bib-0007] Barton, K. E. , & Boege, K. (2017). Future directions in the ontogeny of plant defence: Understanding the evolutionary causes and consequences. Ecology Letters, 20, 403–411. 10.1111/ele.12744 28145095

[fec13234-bib-0008] Bekaert, M. , Edger, P. P. , Hudson, C. M. , Pires, J. C. , & Conant, G. C. (2012). Metabolic and evolutionary costs of herbivory defense: Systems biology of glucosinolate synthesis. New Phytologist, 196, 596–605. 10.1111/j.1469-8137.2012.04302.x 22943527

[fec13234-bib-0009] Bennett, R. N. , & Wallsgrove, R. M. (1994). Secondary metabolites in plant defense‐mechanisms. New Phytologist, 127, 617–633. 10.1111/j.1469-8137.1994.tb02968.x 33874382

[fec13234-bib-0010] Berendsen, R. L. , Pieterse, C. M. J. , & Bakker, P. A. H. M. (2012). The rhizosphere microbiome and plant health. Trends in Plant Science, 17, 478–486. 10.1016/j.tplants.2012.04.001 22564542

[fec13234-bib-0011] Berendsen, R. L. , Vismans, G. , Yu, K. , Song, Y. , de Jonge, R. , Burgman, W. P. , … Pieterse, C. M. J. (2018). Disease‐induced assemblage of a plant‐beneficial bacterial consortium. The ISME Journal, 12, 1496–1507. 10.1038/s41396-018-0093-1 29520025 PMC5956071

[fec13234-bib-0012] Bisseling, T. , & Scheres, B. (2014). Nutrient computation for root architecture. Science, 346, 300–301. 10.1126/science.1260942 25324371

[fec13234-bib-0013] Boege, K. , & Marquis, R. J. (2005). Facing herbivory as you grow up: The ontogeny of resistance in plants. Trends in Ecology & Evolution, 20, 441–448. 10.1016/j.tree.2005.05.001 16701415

[fec13234-bib-0014] Bongers, F. J. , Pierik, R. , Anten, N. P. R. , & Evers, J. B. (2018). Subtle variation in shade avoidance responses may have profound consequences for plant competitiveness. Annals of Botany, 121, 863–873. 10.1093/aob/mcx151 29280992 PMC5906909

[fec13234-bib-0015] Campos, M. L. , Yoshida, Y. , Major, I. T. , Ferreira, D. D. , Weraduwage, S. M. , Froehlich, J. E. , … Howe, G. A. (2016). Rewiring of jasmonate and phytochrome B signalling uncouples plant growth‐defense tradeoffs. Nature Communications, 7, 12570 10.1038/ncomms12570 PMC5155487 27573094

[fec13234-bib-0016] Cates, R. G. (1980). Feeding patterns of monophagous, oligophagous, and polyphagous insect herbivores: The effect of resource abundance and plant chemistry. Oecologia, 46, 22–31. 10.1007/BF00346961 28310621

[fec13234-bib-0017] Cipollini, D. , Walters, D. , & Voelckel, C. (2014). Costs of resistance in plants: from theory to evidence. Annual Plant Reviews, 47 263–307.

[fec13234-bib-0018] Dam, N. M. V. , & Baldwin, I. T. (1998). Costs of jasmonate‐induced responses in plants competing for limited resources. Ecology Letters, 1, 30–33. 10.1046/j.1461-0248.1998.00010.x

[fec13234-bib-0019] de Vries, J. , Evers, J. B. , Dicke, M. , & Poelman, E. H. (2018). Data from: Ecological interactions shape the adaptive value of plant defence: Herbivore attack versus competition for light. Zenodo, 10.5281/zenodo.1468422. PMC6472621 31007332

[fec13234-bib-0020] de Vries, J. , Evers, J. B. , & Poelman, E. H. (2017). Dynamic plant‐plant‐herbivore interactions govern plant growth‐defence integration. Trends in Plant Science, 22, 329–337. 10.1016/j.tplants.2016.12.006 28089490

[fec13234-bib-0021] de Vries, J. , Poelman, E. H. , Anten, N. , & Evers, J. B. (2018). Elucidating the interaction between light competition and herbivore feeding patterns using functional–structural plant modelling. Annals of Botany, 121, 1019–1031. 10.1093/aob/mcx212 29373660 PMC5906910

[fec13234-bib-0022] de Wit, M. , Spoel, S. H. , Sanchez‐Perez, G. F. , Gommers, C. M. M. , Pieterse, C. M. J. , Voesenek, L. A. C. J. , & Pierik, R. (2013). Perception of low red:Far‐red ratio compromises both salicylic acid‐ and jasmonic acid‐dependent pathogen defences in Arabidopsis. The Plant Journal, 75, 90–103. 10.1111/tpj.12203 23578319

[fec13234-bib-0023] Dicke, M. , & Hilker, M. (2003). Induced plant defences: From molecular biology to evolutionary ecology. Basic and Applied Ecology, 4, 3–14. 10.1078/1439-1791-00129

[fec13234-bib-0024] Evers, J. B. (2016). Simulating crop growth and development using functional‐structural plant modeling In Hikosaka K., Niinemets Ü., & Anten P. R. N. (Eds.), Canopy photosynthesis: From basics to applications (pp. 219–236). Dordrecht, The Netherlands: Springer Netherlands.

[fec13234-bib-0025] Evers, J. B. , & Bastiaans, L. (2016). Quantifying the effect of crop spatial arrangement on weed suppression using functional‐structural plant modelling. Journal of Plant Research, 129, 339–351. 10.1007/s10265-016-0807-2 27000875 PMC4850179

[fec13234-bib-0026] Evers, J. B. , Vos, J. , Chelle, M. , Andrieu, B. , Fournier, C. , & Struik, P. C. (2007). Simulating the effects of localized red:Far‐red ratio on tillering in spring wheat (Triticum aestivum) using a three‐dimensional virtual plant model. New Phytologist, 176, 325–336. 10.1111/j.1469-8137.2007.02168.x 17888114

[fec13234-bib-0027] Evers, J. , Vos, J. , Yin, X. , Romero, P. , Van Der Putten, P. , & Struik, P. (2010). Simulation of wheat growth and development based on organ‐level photosynthesis and assimilate allocation. Journal of Experimental Botany, 61, 2203–2216. 10.1093/jxb/erq025 20231326

[fec13234-bib-0028] Feeny, P. (1976). Plant apparency and chemical defense In Wallace J. W., & Mansell R. L. (Eds.), Biochemical interaction between plants and insects (pp. 1–40). Boston, MA, USA: Springer, USA.

[fec13234-bib-0029] Fraser, D. P. , Hayes, S. , & Franklin, K. A. (2016). Photoreceptor crosstalk in shade avoidance. Current Opinion in Plant Biology, 33, 1–7. 10.1016/j.pbi.2016.03.008 27060719

[fec13234-bib-0030] Freckleton, R. P. , & Watkinson, A. R. (2001). Asymmetric competition between plant species. Functional Ecology, 15, 615–623. 10.1046/j.0269-8463.2001.00558.x

[fec13234-bib-0031] Gershenzon, J. (1994). The cost of plant chemical defense against herbivory: A biochemical perspective In Bernays E. A. (Ed.), Insect‐plant interactions (pp. 105–173). Boca Raton, FL:, USA CRC Press.

[fec13234-bib-0032] Gols, R. , Wagenaar, R. , Bukovinszky, T. , van Dam, N. M. , Dicke, M. , Bullock, J. M. , & Harvey, J. A. (2008). Genetic variation in defense chemistry in wild cabbages affects herbivores and their endoparasitoids. Ecology, 89, 1616–1626. 10.1890/07-0873.1 18589526

[fec13234-bib-0033] Heil, M. (2002). Ecological costs of induced resistance. Current Opinion in Plant Biology, 5, 345–350. 10.1016/S1369-5266(02)00267-4 12179969

[fec13234-bib-0034] Heil, M. , & Baldwin, I. T. (2002). Fitness costs of induced resistance: Emerging experimental support for a slippery concept. Trends in Plant Science, 7, 61–67. 10.1016/S1360-1385(01)02186-0 11832276

[fec13234-bib-0035] Heil, M. , Hilpert, A. , Kaiser, W. , & Linsenmair, K. E. (2000). Reduced growth and seed set following chemical induction of pathogen defence: Does systemic acquired resistance (SAR) incur allocation costs? Journal of Ecology, 88, 645–654. 10.1046/j.1365-2745.2000.00479.x

[fec13234-bib-0036] Hemmerling, R. , Kniemeyer, O. , Lanwert, D. , Kurth, W. , & Buck‐Sorlin, G. (2008). The rule‐based language XL and the modelling environment GroIMP illustrated with simulated tree competition. Functional Plant Biology, 35, 739–750. 10.1071/FP08052 32688828

[fec13234-bib-0037] Herms, D. A. , & Mattson, W. J. (1992). The dilemma of plants: To grow or defend. The Quarterly Review of Biology, 67, 283–335. 10.1086/417659

[fec13234-bib-0038] Hikosaka, K. , Anten, N. P. R. , Borjigidai, A. , Kamiyama, C. , Sakai, H. , Hasegawa, T. , … Ito, A. (2016). A meta‐analysis of leaf nitrogen distribution within plant canopies. Annals of Botany, 118, 239–247. 10.1093/aob/mcw099 27296134 PMC4970363

[fec13234-bib-0039] Hirose, T. (2005). Development of the Monsi‐Saeki theory on canopy structure and function. Annals of Botany, 95, 483–494. 10.1093/aob/mci047 15585544 PMC4246794

[fec13234-bib-0040] Hirose, T. , Werger, M. J. A. , Pons, T. L. , & Rheenen, J. W. A. (1987). Canopy structure and leaf nitrogen distribution in a stand of Lysimachia vulgaris L. as influenced by stand density. Oecologia, 77, 145–150. 10.1007/BF00379180 28310366

[fec13234-bib-0041] Izaguirre, M. , Mazza, C. , Astigueta, M. , Ciarla, A. , & Ballaré, C. (2013). No time for candy: Passionfruit (Passiflora edulis) plants down‐regulate damage‐induced extra floral nectar production in response to light signals of competition. Oecologia, 173, 213–221. 10.1007/s00442-013-2721-9 23839264

[fec13234-bib-0042] Johnson, M. T. J. , & Agrawal, A. A. (2005). Plant genotype and environment interact to shape a diverse arthropod community on evening primrose (Oenothera biennis). Ecology, 86, 874–885. 10.1890/04-1068

[fec13234-bib-0043] Koricheva, J. (2002). Meta‐analysis of sources of variation in fitness costs of plant antiherbivore defenses. Ecology, 83, 176–190. 10.1890/0012-9658(2002)083[0176:MAOSOV]2.0.CO;2

[fec13234-bib-0044] Koricheva, J. , & Barton, K. E. (2012). Temporal changes in plant secondary metabolite production: Patterns, causes and consequences In Iason G. R., Dicke M., & S. E. Hartley (Eds.), The ecology of plant secondary metabolites: From genes to global processes (pp. 34–55). Cambridge, UK: Cambridge University Press.

[fec13234-bib-0045] Lankau, R. A. (2007). Specialist and generalist herbivores exert opposing selection on a chemical defense. New Phytologist, 175, 176–184. 10.1111/j.1469-8137.2007.02090.x 17547677

[fec13234-bib-0046] Lankau, R. A. , & Strauss, S. Y. (2008). Community complexity drives patterns of natural selection on a chemical Defense of Brassica nigra. American Naturalist, 171, 150–161. 10.1086/524959 18197768

[fec13234-bib-0047] Lin, T. , Klinkhamer, P. G. L. , & Vrieling, K. (2015). Parallel evolution in an invasive plant: Effect of herbivores on competitive ability and regrowth of Jacobaea vulgaris. Ecology Letters, 18, 668–676. 25958781 10.1111/ele.12445

[fec13234-bib-0048] McKey, D. (1974). Adaptive patterns in alkaloid physiology. The American Naturalist, 108, 305–320. 10.1086/282909

[fec13234-bib-0049] Mommer, L. , Kirkegaard, J. , & van Ruijven, J. (2016). Root–root interactions: Towards a rhizosphere framework. Trends in Plant Science, 21, 209–217. 10.1016/j.tplants.2016.01.009 26832947

[fec13234-bib-0050] Moreno, J. E. , Tao, Y. , Chory, J. , & Ballare, C. L. (2009). Ecological modulation of plant defense via phytochrome control of jasmonate sensitivity. Proceedings of the National Academy of Sciences of the United States of America, 106, 4935–4940. 10.1073/pnas.0900701106 19251652 PMC2660767

[fec13234-bib-0051] Philippot, L. , Raaijmakers, J. M. , Lemanceau, P. , & van der Putten, W. H. (2013). Going back to the roots: The microbial ecology of the rhizosphere. NatureReviews. Microbiology, 11, 789–799. 24056930 10.1038/nrmicro3109

[fec13234-bib-0052] Poelman, E. H. (2015). From induced resistance to defence in plant‐insect interactions. Entomologia Experimentalis Et Applicata, 157, 11–17. 10.1111/eea.12334

[fec13234-bib-0053] Poelman, E. H. , Broekgaarden, C. , Van Loon, J. J. A. , & Dicke, M. (2008). Early season herbivore differentially affects plant defence responses to subsequently colonizing herbivores and their abundance in the field. Molecular Ecology, 17, 3352–3365. 10.1111/j.1365-294X.2008.03838.x 18565114

[fec13234-bib-0054] Poelman, E. H. , & Kessler, A. (2016). Keystone herbivores and the evolution of plant defenses. Trends in Plant Science, 21, 477–485. 10.1016/j.tplants.2016.01.007 26832946

[fec13234-bib-0055] Poelman, E. H. , Van Loon, J. J. A. , Van Dam, N. M. , Vet, L. E. M. , & Dicke, M. (2010). Herbivore‐induced plant responses in Brassica oleracea prevail over effects of constitutive resistance and result in enhanced herbivore attack. Ecological Entomology, 35, 240–247.

[fec13234-bib-0056] Rasmussen, C. R. , Weisbach, A. N. , Thorup‐Kristensen, K. , & Weiner, J. (2017). Size‐asymmetric root competition in deep, nutrient‐poor soil. Journal of Plant Ecology, rtx064 10.1093/jpe/rtx064

[fec13234-bib-0057] Schoonhoven, L. M. , van Loon, J. J. A. , & Dicke, M. (2005). Insect‐plant biology. Oxford, UK: Oxford University Press.

[fec13234-bib-0058] Stam, J. M. , Kroes, A. , Li, Y. , Gols, R. , van Loon, J. J. , Poelman, E. H. , & Dicke, M. (2014). Plant interactions with multiple insect herbivores: From community to genes. Annual Review of Plant Biology, 65, 689–713. 10.1146/annurev-arplant-050213-035937 24313843

[fec13234-bib-0059] Strauss, S. Y. , Rudgers, J. A. , Lau, J. A. , & Irwin, R. E. (2002). Direct and ecological costs of resistance to herbivory. Trends in Ecology & Evolution, 17, 278–285. 10.1016/S0169-5347(02)02483-7

[fec13234-bib-0060] Van Dam, N. M. , & Baldwin, I. T. (2001). Competition mediates costs of jasmonate‐induced defences, nitrogen acquisition and transgenerational plasticity in Nicotiana attenuata. Functional Ecology, 15, 406–415. 10.1046/j.1365-2435.2001.00533.x

[fec13234-bib-0061] Vos, J. , Evers, J. B. , Buck‐Sorlin, G. H. , Andrieu, B. , Chelle, M. , & de Visser, P. H. B. (2010). Functional–structural plant modelling: A new versatile tool in crop science. Journal of Experimental Botany, 61, 2101–2115. 10.1093/jxb/erp345 19995824

[fec13234-bib-0062] Wei, X. , Vrieling, K. , Mulder, P. P. J. , & Klinkhamer, P. G. L. (2015). Testing the generalist‐specialist dilemma: The role of pyrrolizidine alkaloids in resistance to invertebrate herbivores in Jacobaea species. Journal of Chemical Ecology, 41, 159–167. 10.1007/s10886-015-0551-4 25666592 PMC4351440

[fec13234-bib-0063] Weiner, J. (1990). Asymmetric competition in plant populations. Trends in Ecology & Evolution, 5, 360–364. 10.1016/0169-5347(90)90095-U 21232393

[fec13234-bib-0064] Zhu, J. , van der Werf, W. , Anten, N. P. R. , Vos, J. , & Evers, J. B. C. (2015). The contribution of phenotypic plasticity to complementary light capture in plant mixtures. New Phytologist, 207, 1213–1222. 10.1111/nph.13416 25898768

[fec13234-bib-0065] Züst, T. , & Agrawal, A. A. (2017). Trade‐offs between plant growth and defense against insect herbivory: An emerging mechanistic synthesis. Annual Review of Plant Biology, 68, 513–534. 10.1146/annurev-arplant-042916-040856 28142282

[fec13234-bib-0066] Züst, T. , Joseph, B. , Shimizu, K. K. , Kliebenstein, D. J. , & Turnbull, L. A. (2011). Using knockout mutants to reveal the growth costs of defensive traits. Proceedings of the Royal Society of London B: Biological Sciences. 10.1098/rspb.2010.2475 PMC3136827 21270041

