# Supplementary material for: Ecological interactions shape the adaptive value of plant defence: Herbivore attack versus competition for light
Source: Funct Ecol. 2018 Nov 20;33(1):129–38. doi: 10.1111/1365-2435.13234 (PMC6472621; doi:10.1111/1365-2435.13234)
Supplement: Supplementary file 1 [file FEC-33-129-s001.pdf]

Functional Ecology

BRITISH ECOLOGICAL SOCIETY

Herbivore attack versus competition for light shapes the adaptive value of plant defence

Jorad de Vries, Jochem B. Evers, Marcel Dicke and Erik H. Poelman

Plants defend themselves against diverse communities of herbivorous insects. These defences require an investment of limited resources such as carbon-based assimilates obtained through photosynthesis and nitrogen taken up by the root system. Plants compete for these resources with their neighbours and show plastic responses to neighbour presence to enhance their competitive ability. One of the responses upon neighbour detection is a decrease in the plant's ability to defend itself against attackers, presumably to spend the resources otherwise directed towards defence on increasing the plant's competitive ability. Thus the costs associated with an investment in defence are determined not only by the direct metabolic costs, but also the indirect costs that are paid through ecological interactions. The latter have a potentially large impact on the evolution of defensive traits, but have proven difficult to study. In this study we use a three-dimensional modelling approach to quantify the relative impact of the direct and indirect costs and benefits of an investment in plant defence in relation to herbivory and competition for light. Additionally, we evaluated how the plant balances the benefits and costs of defence in the context of herbivory and competition for light. Our modelling results show that the ecological costs of competition for light are detrimental to the fitness of defended plants, as the defended plants have less biomass to capture light with. However, these costs can be balanced by defence reducing the damage done by the herbivores, which is more effective than defence dispersing herbivore damage away from young leaves, and towards older leaves that are less important to plant performance. We conclude that ecological interactions, especially competition for light, have played a major role in the evolution of plant defence.

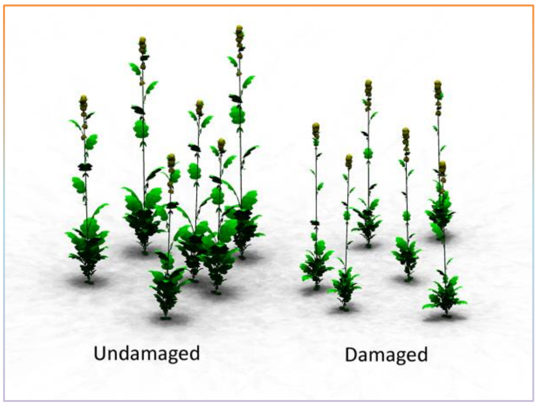

Visual representation of the three dimensional, functional-structural plant model used in the study, showing undamaged and damaged plants. Herbivore damage reduces leaf area, which affects canopy structure and subsequent light climate.
