# Supplementary material for: Ecological interactions shape the adaptive value of plant defence: Herbivore attack versus competition for light
Source: Funct Ecol. 2018 Nov 20;33(1):129–38. doi: 10.1111/1365-2435.13234 (PMC6472621; doi:10.1111/1365-2435.13234)
Supplement: Supplementary file 2 [file FEC-33-129-s002.docx]

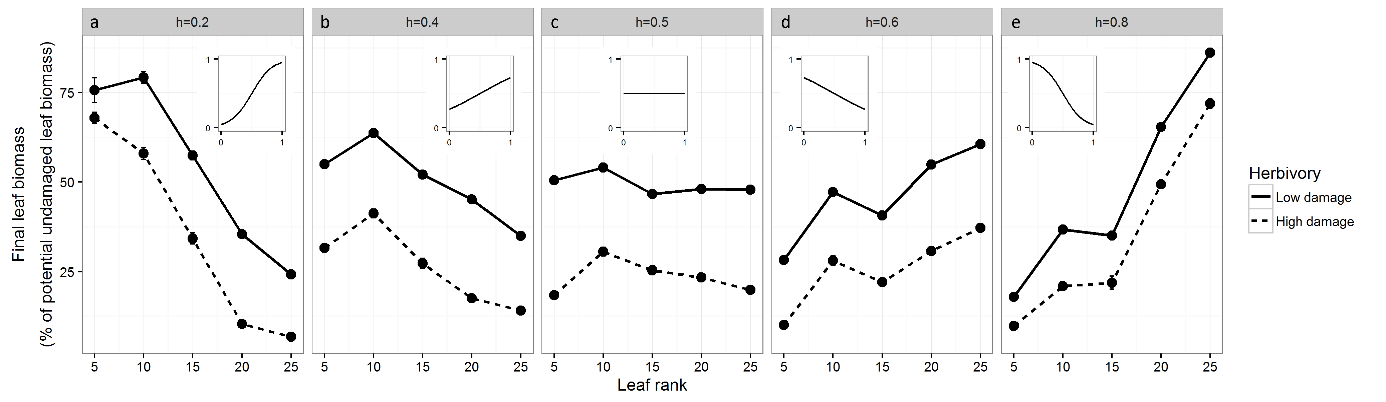

Figure S1. The final leaf biomass as a percentage of potential undamaged leaf biomass (y-axis) of different leaf ranks (x-axis, 5=old leaves at the bottom of the canopy, 25=young leaves at the top of the canopy) for different levels of herbivore damage (low, high) and different herbivore distribution patterns (panels, h in eq. 3) at a 15% plant defence investment. The subplots show the herbivore distribution (y-axis) as a function of relative leaf rank (x-axis, lowest leaf rank = 0, highest leaf rank = 1) for the corresponding value of h. Error bars show standard error of the mean.
